# Guide to Semi-Quantitative Non-Targeted Screening Using LC/ESI/HRMS

**DOI:** 10.3390/molecules26123524

**Published:** 2021-06-09

**Authors:** Louise Malm, Emma Palm, Amina Souihi, Merle Plassmann, Jaanus Liigand, Anneli Kruve

**Affiliations:** 1 Department of Materials and Environmental Chemistry, Stockholm University, Svante Arrhenius väg 16, 114 18 Stockholm, Sweden; loma5202@student.su.se (L.M.); emma.palm@mmk.su.se (E.P.); amina.souihi@mmk.su.se (A.S.); 2 Department of Environmental Science, Stockholm University, Svante Arrhenius väg 8, 114 18 Stockholm, Sweden; Merle.Plassmann@aces.su.se; 3 Quantem Analytics, 510 08 Tartu, Estonia; jaanus@quantem.co

**Keywords:** Ionization, quantification, decision making, NTS strategies

## Abstract

Non-targeted screening (NTS) with reversed phase liquid chromatography electrospray ionization high resolution mass spectrometry (LC/ESI/HRMS) is increasingly employed as an alternative to targeted analysis; however, it is not possible to quantify all compounds found in a sample with analytical standards. As an alternative, semi-quantification strategies are, or at least should be, used to estimate the concentrations of the unknown compounds before final decision making. All steps in the analytical chain, from sample preparation to ionization conditions and data processing can influence the signals obtained, and thus the estimated concentrations. Therefore, each step needs to be considered carefully. Generally, less is more when it comes to choosing sample preparation as well as chromatographic and ionization conditions in NTS. By combining the positive and negative ionization mode, the performance of NTS can be improved, since different compounds ionize better in one or the other mode. Furthermore, NTS gives opportunities for retrospective analysis. In this tutorial, strategies for semi-quantification are described, sources potentially decreasing the signals are identified and possibilities to improve NTS are discussed. Additionally, examples of retrospective analysis are presented. Finally, we present a checklist for carrying out semi-quantitative NTS.

## 1. Different Strategies

A general workflow for non-targeted screening (NTS), as described by Hollender et al. [1], includes representative sampling followed by enrichment suitable for the sample matrix. E.g., for water matrices, solid-phase extraction (SPE) is ordinarily used; however, in order to not lose compounds of interest, the stationary phase used should be chosen with care [1,2]. For separation and analysis of the sample, liquid chromatography electrospray ionization high resolution mass spectrometry (LC/ESI/HRMS) is utilized. Commonly, the chromatographic separation is performed in reversed phase, and the HRMS is run in full scan mode. After collecting the data, the next step is peak detection and grouping of peaks related to the same molecular structure, and also comparison of sample peaks with peaks from compounds present in blanks [1,2,3]. This step can often be semi-automated, using either the vendor, third-party or open-source software (e.g., Thermo Scientific^TM^ Compound Discoverer^TM^ software, [4] envipy [5] from EAWAG or MZmine [6]). Still, even after this data pre-processing step, there are usually too many peaks left for all to be confidently (to level 1 and 2 according to the Schymanski scale) identified, [7] thus the peaks are prioritized before the ones with highest priority are fully identified with analytical standards [1,3]. Every so often, we simply prioritize the compound for which we have information—e.g., if standards are available, if they are toxic or known to be very abundant or persistent. It might also be of interest to prioritize compounds with many transformation products (TPs), as the TPs can sometimes be more toxic [8] or more abundant [8,9] than the parent compound. To accurately prioritize the peaks, it is desired to know the concentrations of the compounds, however, the ionization efficiency (*IE*) of different compounds varies tremendously in ESI, making it inappropriate to only compare the intensities in the spectrum for prioritizing. Instead, semi-quantitative approaches should be included in the NTS workflow before prioritizing the peaks.

As mentioned, *IE* varies significantly in the ESI source, depending on the properties of the compound itself but also the properties of the sample matrix and the eluent used [10,11]. While the ionization efficiency is influenced by many factors two general rules to consider are: (1) in positive ionization mode stronger bases and in negative ionization mode stronger acids, and (2) more hydrophobic compounds, have higher ionization efficiency. Additionally, the increased amount of organic solvent will increase the *IE*, since the evaporation rate of the droplet will increase [11]. Therefore, compounds eluting later from reversed phase chromatography tend to have higher ionization efficiency values. Moreover, the pH of the mobile phase in combination with the acidity/basicity of the compound also have great influence of the *IE*: compounds with acidic moieties usually ionize more efficiently in basic environments in negative ESI mode (ESI-), while compounds with basic moieties usually have higher *IE* in acidic mobile phase in positive ESI mode (ESI+) [12,13]. Many additional factors impacting ionization efficiency have been studied previously relating to compound structure, mobile phase, and instrument parameters. 

A measurement that is closely related to the ionization efficiency of a compound is the response factor (RF), the ratio of the detected peak area and the concentration of the compound (Equation (1)). This ratio is important in some of the semi-quantification strategies presented below.

(1) $$RF=\frac{peak\;area}{concentration}$$

As the main focus of this tutorial is on the practical implementation of semi-quantification, we will not go too deep into details regarding ionization efficiency and kindly direct the interested reader towards excellent reviews/studies published previously, e.g., for compound properties see Kruve [2] and Cech and Enke [12]; for mobile phase properties Kostiainen and Kauppila [14] and Kiontke et al. [15]; for source parameters Page et al. [16] Pieke et al. [17] and Espinosa et al. [18].

### 1.1. Structurally Similar Compounds

One approach for semi-quantification is to use standards with structural similarities to the unknown compound(s). This approach however is not true non-target screening but rather suspect screening, since a tentative structure of the unknown, or suspect, is needed. The assumption is that the response of the structurally similar compound will be similar to that of the suspect, and therefore, the RF of the similar standard can be used to estimate the concentration of the unknown (Equation (2)).

(2) $$c_{suspect\;compound}=\frac{{peak\;area}_{suspect\;compound}}{{RF}_{similar\;compound}}$$

An online tool [19,20] is available from the University of Athens to search for structurally similar compounds based on 2D similarities, e.g., number of similar functional groups and distance between functional groups [21]. The suspects are compared with compounds from NORMAN SusDat Database, [22] with similarity down to 5%. The similarity is measured as Tanimoto similarity of substructure-based fingerprints. However, the similarity metric does not yet take into consideration if the properties are relevant or not for ionization efficiency in electrospray.

One special class of compounds are transformation products. Many compounds, e.g., pesticides and pharmaceuticals, degrade naturally in the environment, giving rise to transformation products [3,9,23]. TPs can also be formed by chemical degradation, e.g., via oxidation processes in wastewater treatment plants [3,24]. Although TPs can be more persistent, [9] abundant [3,9] and/or toxic [8,24] than the parent compound, they are rarely included in environmental targeted analysis, e.g., in water protective plans. This is primarily because of the unknown toxicity [9] and/or structure [2] of many TPs. However, it is possible to semi-quantify transformation products based on the parent compounds RF, in an analogous approach as the similar compound approach described above (Equation (3)).

(3) $$c_{TP}=\frac{{peak\;area}_{TP}}{{RF}_{parent\;compound}}$$

This strategy is based on the presumed structural similarity between TPs and their parent compounds; however, sometimes the TPs have lost functional groups that largely affect the ionization efficiency. As an example, we can look at atrazine and some its known degradation products, to see how the structure changes, see Table 1. We can compare how the similarity score, obtained from the University of Athens online tool, [19,20] changes with the structure. As seen, the similarity decreases drastically as the structural differences increase. Surprisingly, the hydrophobicity of the compound does not seem to influence the overall similarity, as the log*P* varies quite a lot between the parent and TPs while the similarity scores remain high. This, however, makes the approaches utilizing structurally similar compounds prone to higher errors, as the hydrophobicity is known to have a high impact on the ionization efficiency [11,25]. The decrease in response factors with the decrease in similarities further indicates that such approaches, although easy to use, might give higher errors than alternative approaches.

Additional limitations in the transformation product quantification with parent compounds are that sometimes, the parent compound and transformation products cannot be detected with the same analysis mode. For example, chlorothalonil is not detected in LC/ESI/HRMS while the corresponding TPs are well detectable. Furthermore, while flufenacet is usually analyzed in ESI+, its transformation products flufenacet-OXA and flufenacet-ESA are analyzed in negative ionization mode [9]. 

Theoretically, this approach works for all compounds that have either structurally similar compounds or a parent compound. Although the parent compound-TP approach is usually applied in environmental analysis, it can also be used to estimate concentrations of metabolites in, e.g., biological samples [27].

### 1.2. Close Eluting Compounds

Another strategy for semi-quantification of unknowns is to use the RF of the compound with known concentration (internal standard) that is eluting closest to the unknown compound in the chromatogram. This strategy was proposed by Pieke et al. [17] and is based on the assumption that compounds that elute close in time in reversed phase LC, i.e., compounds that share similarities in chromatographic properties, will also have similar RF. The factors influencing retention in reversed phase LC are similar to those influencing *IE*, e.g., the polarity of the compound and its acid/base properties. Thus, the concentration of the unknown compound can be estimated (Equation (4)).

(4) $$c_{unknown\;compound}=\frac{{peak\;area}_{unknown\;compound}}{{RF}_{closest\;eluting\;compound}}$$

How many internal standards are needed depends on the sample, i.e., on how many unknown compounds that it contains. However, a good rule of thumb is to have both early, middle, and late eluting standards with known concentration, since we do not know beforehand where our unknown compounds will elute. Because of the large variation in *IE*, which strongly affects the RF, it has also been proposed to include internal standards that covers a wide range of response factors [17]. This strategy is in theory applicable for any class of compounds that can be analyzed with LC/MS, in both ESI+ and ESI-. However, to our knowledge it has not yet been validated for a wide range of compounds. 

For this approach, the chromatographic conditions also play an important role. In some cases, the closest eluting compound may not be the same at different pH conditions as the compound’s retention time changes with protonation and deprotonation. We evaluated the impact of chromatographic conditions for a set of 81 compounds with mobile phases of three different pHs. For two compounds, metsulfuron-methyl and valsartan, drastic changes in retention time were observed. This also changes the closest eluting compound and thus the accuracy of the response factor prediction. For example, metsulfuron-methyl and its closest eluting compound prometryn have response factor ratio of 0.692 at pH 2.7 while at pH 5.0 the closest eluting compound is 2-napthoic acid and the response factor ratio is 857, as shown in Table 2. This variation indicates that retention time and ionization efficiency are influenced by somewhat different factors. 

### 1.3. Predicting Ionization Efficiency

The last approach for semi-quantification of suspects/unknowns is to use machine learning to train models to predict the *IE* of the compounds. Factors that influence the ionization efficiency has been widely studied by many researchers, [12,28,29] and there are also many papers describing models to predict the *IE* [25,30,31]. However, most earlier studies and models have focused on either ESI+ or ESI-, and on one class of compounds. A comprehensive comparison of previous *IE* studies has been done by Liigand et al. [32] Recently, we have presented an *IE* prediction model that works for positive and negative ionization mode [10]. Data from 106 eluent compositions with 353 compounds in positive mode, and 33 eluent compositions with 101 compounds in negative mode, was used. The model utilizes random forest regression to predict the ionization efficiency based on 2D PaDEL descriptors (450 in ESI+ and 145 in ESI-) together with eluent descriptors. Because the ionization efficiency can differ so drastically between different instruments, all *IE* measurements used for the model development were performed relative to tetraethylammonium and benzoic acid for ESI+ and ESI-, respectively. Furthermore, all *IE* values were unified by performing linear regression between the measurements from two different instruments, to transform the predictions to instrument specific values, and thereby making the results comparable between different instruments [10]. 

The obtained *IE* predictions are related to a predicted RF (Equation (5)), which is used to calculate the concentration of the suspect compound (Equation (6)).

(5) $${logRF}_{pred}=slope\times {logIE}_{pred}+intercept$$

(6) $$c_{suspect\;compound}=\frac{{peak\;area}_{suspect\;compound}}{10^{logRF_{pred}}}$$

A commercial online tool [33] provided by Quantem Analytics is available to semi-quantify suspect compounds based on the *IE* prediction model. It requires information about analysis conditions (ionization mode, eluent composition, gradient program) and a csv file with SMILES, retention time and signal obtained, as well as at least 5 compounds with known concentration. These compounds with known concentration are used to calibrate the predictions made by the tool.

Currently, the *IE* prediction model described here is only applicable for compounds that form [M+H]^+^, [M]^+^, and [M−H]^-^ ions, as such compounds was used to train the model. Therefore, some compounds, e.g., biomolecules like sugars, are not suitable to semi-quantify with this approach, as they predominantly form adducts [10]. Additionally, any machine learning model can only be applied to the compounds structurally similar to the ones used in training the models. Therefore, the application range depends on the concrete model and its basis. The *IE* prediction model published by Liigand et al. [10] was trained and tested on a large range of compound classes, e.g., benzenoids, organo-heterocyclic compounds, organic acids, etc., and therefore covers a wide chemical space. It has also been validated on pesticides and mycotoxins.

The second semi-quantification approach is the only true non-targeted approach, as no tentative structure of the suspect compound is required as opposed to the other strategies. However, the use of high resolving instruments has improved the mass accuracy obtained, and thereby made it easier to assign a tentative structure based on the exact mass and MS/MS fragmentation spectra [1,10,34]. 

Most of the semi-quantification strategies described here are relatively straight forward and easy to use. Therefore, we suggest investigating if combinations of the approaches could improve the results and decrease the errors related to the predicted concentrations.

## 2. Sources Decreasing the Signal of the Molecular Ion

Any of the described approaches can only account for the factors it has been based on. Quantification models that are trained to account for the differences in the ionization efficiency of different compounds, for example, will not be able to account for the factors in other parts of the analysis that also influence the signal of the compounds. In non-targeted LC/HRMS many sources for signal variation occur, starting from the very beginning of the workflow to the end. In sample preparation, different compounds may have very-different recoveries while chromatography may cause peak artifacts. Additionally, the softness of ionization conditions and ion transportation may influence the intensity of the signals observed in the final spectrum. Finally, the automatic integration has to be accurate to obtain reliable results.

### 2.1. Sample Preparation

The relatively low concentrations of compounds in the samples of interest often require preparing the samples before analysis. Many samples, including biological tissues, soil, or other heterogeneous non-liquid samples, also require sample preparation to obtain a liquid sample. The methods used for preparing water samples prior to non-targeted LC/HRMS analysis may involve sample filtration, [35] vacuum-assisted evaporative concentration, [9] SPE, [36] liquid-liquid extraction (LLE), [37,38] or similar. While filtration can be sufficient for preparation of drinking water, more complicated water samples such as wastewater are often extracted with SPE or LLE. LLE is mostly used for preparing samples for GC/MS analysis but can also be used for preparing water samples for LC/HRMS [37]. In LC/ESI/HRMS, SPE is the most widely used sample preparation strategy, whereby very-different protocols are used. Hydrophilic-lipophilic balance, cation exchange, anion exchange, reversed-phase or mixed-mode SPE sorbents are the most-commonly used, either alone [39,40] or in combination [36].

In sample preparation, some compounds will be extracted while other compounds will be removed due to the selectivity of the SPE procedure. This will determine which compounds can be detected with the NTS methods and directly influences the scope of the methods. However, extraction also influences quantification. Even if extracted, the recovery of different compounds varies. Schultze et al. [41] found reversed-phase SPE to yield superior recoveries and detection limits over anion- and cation exchange SPE for water samples. However, only 159 of 251 studied analytes yielded recoveries within the acceptable range of 60 to 123%. Even more so, lowest recoveries that still enabled analyte detection were close to 1%. From the quantification point of view, a recovery of 50% will end up with a quantification error of a factor of 2, while recovery of 1% results in an error of a factor of 100. These effects are much more dramatic in comparison to targeted methods, as no isotopically labelled internal standards are available for correcting with the poor recovery. 

**Figure 1:**
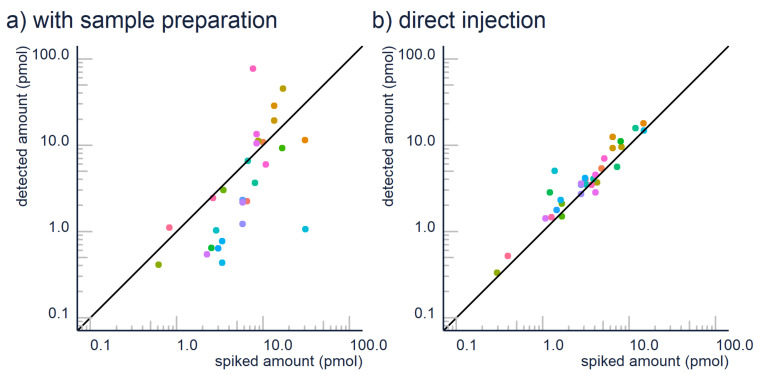
Sample preparation can have a devastating effect on the quantification accuracy as seen here: (**a**) quantification results for contaminants analysis with SPE extraction and (**b**) without SPE extraction, but with a high injection volume. Color corresponds to compound, and both analyzes come from the average of three replica.

Therefore, we suggest (1) avoiding extraction where possible, and (2) further research on extraction efficiency. In some cases, it is possible to avoid sample preparation. For example, the detection limits of contemporary LC/ESI/HRMS devices often reach ng/L levels, so many environmental contaminants can be analyzed directly from the drinking and surface water samples. In our experiences, the matrix effect caused by remaining “matrix” components is negligible compared to the losses in extraction efficiency. This can be also seen in Figure 1, where we have compared the quantification accuracy for 30 micropollutants with a generic hydrophilic-lipophilic balance SPE vs. direct injection. It is clear that many compounds have poor recoveries with SPE; therefore, the estimated concentrations may be an order of magnitude lower than the actual concentrations. 

However, in some cases the sample preparation has to be used. In such cases a generic sample preparation, proven in the literature, is suggested [42]. It is advantageous to use a sample preparation method for which recoveries for many different compounds have been previously determined and are publicly available. This will allow to evaluate the suitability of the extraction method in the context of quantification. A word of caution is needed here, although informative, the recoveries determined for test compounds do not have to apply for the compounds detected with non-targeted analysis. Especially if the physicochemical properties are significantly different. 

Another possibility is to account for the differences in extraction efficiency with modeling approaches. The current problems here are that very few datasets of recoveries are available, and the extraction conditions used in SPE vary tremendously. This includes both the choice of SPE stationary phase, but also solvent. In contrast to electrospray ionization efficiency, the choice of solvents can impact the extraction recoveries of different compound groups very differently, while in electrospray many effects are common to all compounds and can be generalized. To overcome the sparsity of data needed for efficient modeling, both standardization of the sample preparation methods as well as common efforts in collecting data are required.

### 2.2. Chromatography

**Figure 2:**
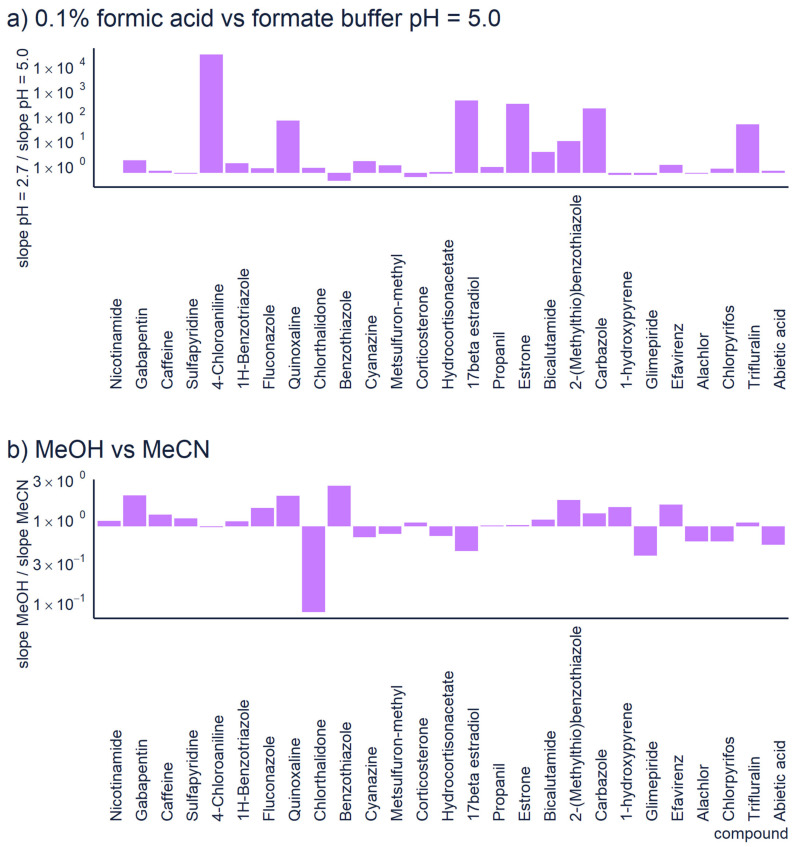
The comparison of sensitivity obtained with (**a**) 0.1% formic acid vs. 10 mM formate buffer at pH = 5.0 and (**b**) using methanol or acetonitrile as organic solvent. In all cases reversed phase chromatography with the same gradient program was used (linear gradient from 5% to 100% of organic modifier over 15 min).

In non-targeted LC/HRMS, the quantification of unknown compounds is highly affected by the chromatographic conditions [13,14]. For example, the mobile phase pH influences the sensitivity of positive ionization mode; acidic conditions show better sensitivity towards basic compounds because their p*K*_a_ values are higher than the pH, and protons are abundant. The sensitivity of analysis decreases at higher pH because the protonation is less efficient, see Figure 2a. However, sometimes the acidic mobile phase can decrease retention and symmetric peak shape [43]. In basic conditions, the “wrong-way-round” ionization could improve the sensitivity towards basic compounds using ammonium formate [44]. In addition, a recent study reported that basic mobile phase significantly improved the ionization of both basic and acidic pharmaceuticals in positive mode [43]. 

The ionization efficiency of a compound is also be affected by acids and buffers. It is widely discussed that trifluoroacetic acid causes ionization suppression in electrospray due to formation of gas phase ion pairs [45,46]. In addition, formate and acetate have higher ability to form ion-pairs compared to fluoride and therefore they lead to lower ionization efficiencies compared to buffer containing fluoride [47]. Ojakivi et al. [48] has observed that acids provide higher ionization efficiencies compared to buffers containing ammonium ions at same pH. This was explained by the fact that ammonium ions have a higher tendency to occupy the charged surface of droplet.

Additionally, the organic solvent and its content may influence the ionization of compounds in reversed phase chromatography. The sensitivity generally increases with higher amount of organic modifier. Polar compounds, which are less retained by the column, elute in more water rich mobile phase and therefore their ionization may be low. Although, at equal organic modifier concentration, methanol and acetonitrile yield indistinguishable ionization efficiency values, [49] the organic solvent and its content may influence the ionization of compounds in reversed phase chromatography. Methanol has a lower elution strength compared to acetonitrile, which allows compounds to elute later at a higher organic solvent content; therefore, the ionization of compounds is improved [14]. Moreover, methanol might improve the peak shape and symmetry of basic compounds [14]. In Figure 2b, we have compared the sensitivity of 27 compounds analyzed with methanol vs. acetonitrile as organic modifier. For this dataset no one organic modifier had significant advantages over another. The differences in sensitivity are generally small, less than 3×. However, one compound shows 10 × higher sensitivity with acetonitrile. This comparison also accounts for the different retention times with the two modifiers. In case of non-targeted analysis, one still needs to understand the chemical space they are most interested in. Although acetonitrile is a widely used organic modifier in reversed phase LC, it may not be suitable in all circumstances. E.g., Colizza et al. [50] have observed that in case of cyclic peroxides, already small amount of acetonitrile in the eluent composition results in strong ion suppression.

The use of binary mobile phase may also influence the quantification of compounds due to peak artifacts in reversed phase LC [51]. The injection of sample can introduce disturbance of the equilibrium state in the column between the mobile and stationary phases. The analytes flow through the column with different velocity than the original equilibrium. Thus, the chromatographic system sets a new equilibrium via a relaxation process, which leads to additional signals in the chromatogram called system or solvent peak [52]. The quantification and identification of unknown compounds become more complicated due to the presence of peak artifacts in the chromatogram.

In general, we have observed that compounds with higher ionization efficiency have lower prediction errors in semi-quantification. This means that it is favorable to use a mobile phase with a pH that aids the ionization in the mode one is using. Thus, in positive mode, mobile phase with lower pH will likely give lower prediction error and mobile phase with higher pH is more likely to give lower prediction errors in negative mode.

We strongly encourage community to use generic standardized methods to collect standardized datasets for future model development of any kind/phenomenon in the benefits of the community and society. In a recent quality assurance paper, [53] every participant in an interlaboratory comparison used slightly different gradient, which makes it unnecessarily complicated to use these datasets for generalization training, i.e., retention time models. For example, Domingo-Almenara et al. [54] have done tremendous work and collected reversed phase retention times for more than 80,000 compounds. However, to use their data in other laboratories, the gradient and solvent needs to match between laboratories. This becomes less feasible when the database has been collected with niche mobile phases or exotic gradients, or if laboratories intend to use very specific chromatographic conditions. Therefore, standardization of chromatographic conditions is highly needed. While comparing the gradient programs used when collecting MassBank data, many different gradients have been used. However, generally 0.1% formic acid is used as water phase and the gradient starts from 1 to 13% of water phase. To generalize these methods, we recommend using 0.1% formic acid in ESI positive mode and ammonium hydroxide or ammonium formate pH = 8.0 in ESI negative mode. In both cases a linear gradient from 5 to 100% of organic modifier over 15 min for a 10 cm C18 column with 3 µm particle size has proven generic. Currently more ionization efficiency data have been collected with acetonitrile than with methanol, with the modeling results for acetonitrile having higher confidence. As a result, we suggest using acetonitrile as the organic modifier in reversed phase chromatography if semi-quantification is intended. 

### 2.3. Ionization Conditions and Ion Transport

Different ionization sources have different softness. Some sources cause extensive fragmentation while other sources facilitate the formation of the molecular ion only. It is suggested that fragmentation occurs after the ejection of the molecular ions from charged droplets to the gas phase. This fragmentation can be brought about in the source, but also while entering the vacuum region of the mass spectrometer. Due to the fact that fragmentation yields are strongly different from source to source and compound to compound, all gas phase ions formed from the compound of interest needs to be accounted for.

In addition to ESI source design and conditions, ion optics and/or use of ion mobility before the mass analyzer may also play a role [55]. Seo et al. [56] observed that protonated *ortho*-aminobenzoic acid is very fragile and proposed that collision with N_2_ in the ion mobility cell or source region provides enough energy for efficient fragmentation. We have a similar observation for *ortho*-aminobenzoic acid when using Agilent instrument with ionFunnel ion focusing. An abundant peak at *m/z* = 120, corresponding to water loss, is observed in the mass spectrum, but only a small or no molecular ion peak.

One remaining challenge in NTS is identifying which fragments have been formed from specific compounds. Most data treatment software and packages do not enable automatic grouping of in-source fragments together with the molecular ion. This is also understandable, as it is essentially impossible to automatically identify which ions simply have very close retention times, and which belong to the same compound. The option here is to analyze fragmentation spectra. The fragments formed in-source and fragments formed from the same compound in MS/MS are the same. Therefore, knowing the fragment ions from the data independent or data dependent acquisition experiments will allow identifying the in-source fragments too. These possibilities, however, have not yet been automated in current software. 

### 2.4. Data Processing

Software used in non-target screening mostly does a good job when it comes to integrating peaks. In some software, the integration algorithm can be chosen by the user depending on the chromatography (e.g., MZmine [6]), while in others, the software itself decides which the best algorithm to use is (e.g., Compound Discoverer [4]). However, many purely non-targeted processing software do not allow changing or deleting peak integrations after processing. And in some instances, like peak splitting or long tailing/fronting, only parts of the peaks might be integrated. Additionally, the alignment between samples can for some compounds with unstable retention times be difficult. Both a wrong integration and a bad alignment can result in a feature being listed twice with different retention times. This means that one always needs to check the integrations of those substances being used during semi-quantification. A solution for substances with bad integration is then to switch to a quantification software to integrate those peaks and, if necessary, do the integration manually.

## 3. Improving the Performance

When estimating the concentrations, it is often believed that the accuracy of the estimation will depend on two things: (1) which features are being used, and (2) what kind of model is selected. In many ways this is true. Firstly, the features must be able to describe how well the analytes will ionize in the source, and secondly, the model must be suitable for the type of data that we have. However, making a better model is not the only way to improve the prediction accuracy. In fact, decisions can be made already in the analysis step to get more accurate estimations of the concentrations.

### 3.1. Increasing the Accuracy by Combining Positive and Negative Mode

Generally, when doing an experiment, be it targeted or non-targeted, one of the first things to decide is how the analytes will be detected. In case of non-targeted analysis with LC/ESI/HRMS, the decision shifts to whether the analysis should be carried out in positive or negative mode. For ESI+, the main advantage is that it can detect more compounds, [57] which is especially beneficial in NTS as the compounds present in the sample are unknown. ESI- on the other hand has the advantage of lower noise levels, [58] and in some cases also has higher sensitivity, which allows for lower detection limits [57]. Different compounds will also ionize to different extent in the different modes depending on several factors. E.g., acids generally ionize better in negative ESI mode, while bases ionize better in positive ESI mode due to their ability to become deprotonated and protonated [58]. For non-target analysis, however, we might not know if the compounds are acids or bases. So how do we choose which mode to use?

One possible answer to that question is that we do not choose one mode, but instead run our analysis with alternating positive and negative mode. Even better would be to run the analysis once in positive mode with an acidic eluent, [13] and once in negative mode with a basic eluent, [59] as different eluents are beneficial in the different modes. Doing the analysis this way has multiple advantages: one major being the simultaneous detection of compound that ionize only in positive or negative mode. This means that more compounds in general are detected, giving a more complete picture of what is present in the sample. Another advantage is the confidence in the quantification results. For the compounds that ionize in both positive and negative mode, the ionization efficiency in both modes can be modeled [10] and used for estimating of the concentration. These two estimated concentrations can then be compared, and if they are similar, it will give more confidence to the accuracy of the models’ predictions. In addition to being able to compare the two obtained concentrations, both predictions could also be combined. 

We have tested four approaches to obtain predicted concentrations using the model developed by Liigand et al. [10] on 39 compounds that are detected in both positive and negative mode at pH = 2.7 and 10, respectively. The alternatives for quantification are to use the concentrations obtained from either (1) positive or (2) negative mode, (3) using the mean of the concentrations from the two modes, or (4) selecting the concentration from the mode with the highest peak area/sensitivity. 

The predictions were done by first predicting the ionization efficiency for each compound in the two modes, followed by calculating the predicted concentrations in the two separate modes (equations 4 and 5). The predicted concentrations for the two other approaches, the mean and the choice based on peak area, were calculated with Equations (7) and (8).

(7) $$c_{mean}=\frac{c_{positive}+c_{negative}}{2}$$

(8) $$c_{choice}=\left\{\begin{array}{l} c_{positive},\;\mathrm{if}\;{peak\;area}_{positive}>{peak\;area}_{negative}\\ c_{negative},\;\mathrm{otherwise}\; \end{array}\right.$$

The prediction errors were calculated by comparing the predicted concentrations with the known concentrations (Equation (9)).

(9) $$prediction\;error=\left\{\begin{array}{l} \frac{c_{predicted}}{c_{known}},\;\mathrm{if}\;c_{predicted}>c_{known}\\ \frac{c_{known}}{c_{predicted}},\;\;\mathrm{otherwise}.\; \end{array}\right.$$

**Figure 3:**
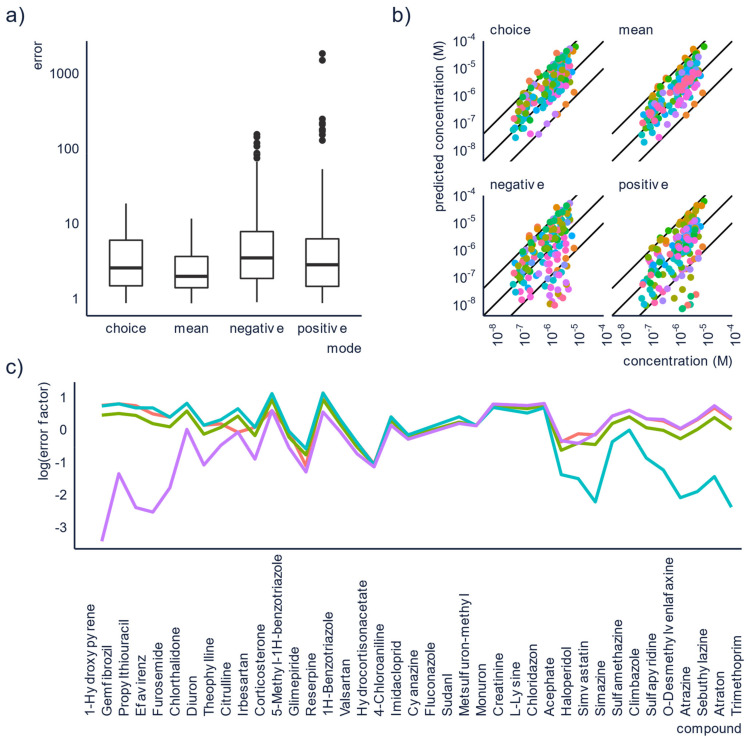
Running the analysis in both ESI+ and ESI− and combining the results appear to yield lower prediction errors. Here we see (**a**) boxplot of the errors of the concentrations predicted with the four different approaches, (**b**) scatter plot of the known concentration vs. the predicted concentration, colors are corresponding to different compounds. The outer lines show an error of a factor of 10 and the middle line shows the ideal prediction, and (**c**) plot of the error for each of the compounds in each mode. The purple line shows positive mode, the blue line shows negative mode, the red line shows the mean approach, and the green line shows the choice approach.

The obtained errors for both the individual concentrations, and the combined approaches were then compared and a statistically significance (Kruskal-Wallis test which gave a *p*-value of 1.073 × 10^−5^. This suggests that at least one of the approaches gives significantly different errors from the other approaches. This was then further investigated with a Dunn’s test which showed that all approaches differ in their prediction accuracy at the 95% confidence level) was observed; therefore, mean and median errors were calculated for all approaches. The lowest mean errors were obtained for the mean and choice approaches, 3.6 and 4.6, respectively. These are significantly lower than the mean errors for the concentrations obtained from the individual modes, which were 40.0 for ESI+ and 12.8 for ESI-. Similarly, the median errors were also lower for the approaches using the combined concentrations, although they were fairly low for the individual modes too. In positive mode, the median error was 3.3 and in negative mode it was 4.0. For the combined modes the corresponding values were 2.3 and 2.9 for the mean and the choice approach, respectively. In Figure 3a,b, the approaches of using positive or negative mode individually can be seen to yield much higher errors for some of the compounds, compared to the combined approaches.

The reason for this improvement in the prediction accuracy can be explained from how ensemble models improve prediction accuracy. For the mean approach, when the prediction accuracy is poor in one mode, this can be partially compensated for by a better prediction accuracy in the other mode. For the choice approach on the other hand, we have observed that the prediction accuracy tends to be poorer for compounds with a low response factor. Consequently, by always relying on the mode with the highest peak area, and thereby the highest response factor, some of the worst errors should be avoided. This is visualized in Figure 3c, where compounds with overpredicted concentrations in positive mode will generally be underpredicted in negative mode and vice versa. 

### 3.2. Dilution of the Sample

To do semi-quantification of the compounds in a sample, what is really done is an estimation of how well the compound will ionize in the mass spectrometer. Or, in other words, an estimation of the slope of its hypothetical calibration curve. This hypothetical slope is then used to calculate the concentration of the analyte. However, semi-quantification approaches do not tell the linear range of the hypothetical calibration curve. Therefore, if the measured concentration does not fall within the linear range of the calibration curve, we risk underestimating the concentration. 

One way to remedy this is to analyze dilutions of the sample and verify linearity. Instead of only analyzing the same sample in two or three replicas, different dilutions should also be analyzed, e.g., 1×, 2× and 5×. Later, the semi-quantification approach would be applied independently for each of the dilutions and corrected for the dilution made. The comparison of these concentrations reveals which compounds in the sample have concentrations that fall outside the linear range of the calibration curve. The concentrations of these compounds should be estimated from one of the more diluted samples. For example, if the 1× dilution yields a significantly lower predicted concentration we can suspect exceeding upper limit of linearity. However, if the concentrations predicted for 2× and 5× dilutions match, these results can be averaged to obtain a final prediction.

**Figure 4:**
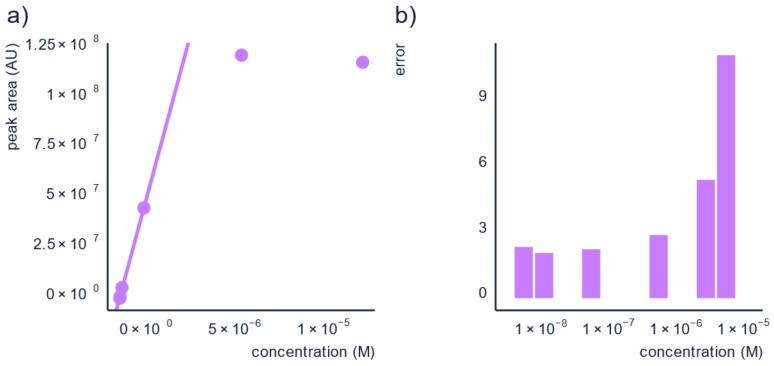
Performing semi-quantification on diluted samples within the linear range reduces the prediction errors, as seen here: (**a**) the calibration curve for pyridaben, and (**b**) bar-plot of the errors for each point in the calibration curve.

To illustrate this, we used data from Wang et al. [60] and calculated the prediction errors of the concentration for pyridaben in a solvent matrix which had concentrations falling outside the linear range. The calibration curve and prediction errors are shown in Figure 4. The prediction errors were found to be between 2.1× and 2.9× for the dilutions in the linear range, whereas the concentration above the linear range had prediction errors of a factor of 5.4× and 11.1×. Briefly, by making sure the semi-quantification is performed in the linear range, the prediction error can be reduced.

### 3.3. Quality Control

Quality control (QC) measures are as important in non-targeted screening as they are in targeted analysis and can shed light on human errors, malfunctioning of instrument, or severe contamination of the instrument. Our suggestions are to use the full potential of such measures to ensure high quality of the semi-quantification. 

Firstly, technical replicates are a very-good measure to pinpoint human errors while handling samples. We suggest analyzing samples in 2, or if possible 3, replica. This can be advantageously combined with the analysis of different dilutions of the samples, described in more detail in Section 3.2. However, technical replicates can unfortunately not correct for systematic effects, such as poor recovery in sample preparation, or overlooking fragments formed in-source. Therefore, it must be stressed that agreement between technical replicates does not necessarily translate to high accuracy in semi-quantification, but rather assures that no fatal errors have occurred during sample processing or analysis. 

Secondly, analysis of dirty samples over a long period of time can make the ion optics of the instrument very dirty, and thereby reduce the sensitivity of the instrument [61]. Therefore, the same sample analyzed at the beginning and at the end of the sequence may yield significantly different peak areas. A possibility to overcome this is by spiking the samples with the compounds used for transferring predicted ionization efficiency values to the instrument specific response factors. The response factors of these calibration compounds will be affected by the cleanliness of the instrument in the same manner as the unknown compounds, thus changes in instrumental signal are automatically accounted for. 

Additionally, QC samples, i.e., analysis of samples spiked with known concentrations of analytes, can be used to evaluate the stability of the instrument, both the within-day and day-to-day performance, and can also account for drift in signal. [62,63] This is especially important during long analyzes, as signal have been shown to drift over ±25% during a 12 h run [64]. For more details on quality control in non-targeted screening see recent reviews by Knolhoff et al. [65] and Schultze et al. [66]. 

## 4. Opportunities Beyond Instrumental Analysis

Contributing to non-targeted analysis does not only mean needing to acquire new and expensive instrumentation and run new samples. Retrospective analysis as well as data repositories give opportunities to contribute without running any new experiments. 

Community is fortunately already starting to store collected data for re-analyzes in the future with more advance tools and more knowledge. NORMAN digital sample freezing platform [67] is one of these platforms giving the possibility to run new quantification and/or identification experiments on already collected samples. The NORMAN community have set a nice example to collect and record as comprehensive data and metadata as possible, making it available for the next research ideas. Other communities that use non-targeted analysis, e.g., metabolomics, also have open data repositories (e.g., Metabolights [68], Metabolomics Workbench [69], GNPS MassIVE [70]) for collecting raw data files.

Based on the data from such platforms, semi-quantitation makes it possible to discover pollutants in environmental samples, or biomarkers in biological samples, that have previously flown under the radar in methods based solely on signal. Revealing the concentration of these compounds can prove their importance, even if their peak area is small, or allow comparison with samples collected and measured later. 

**Figure 5:**
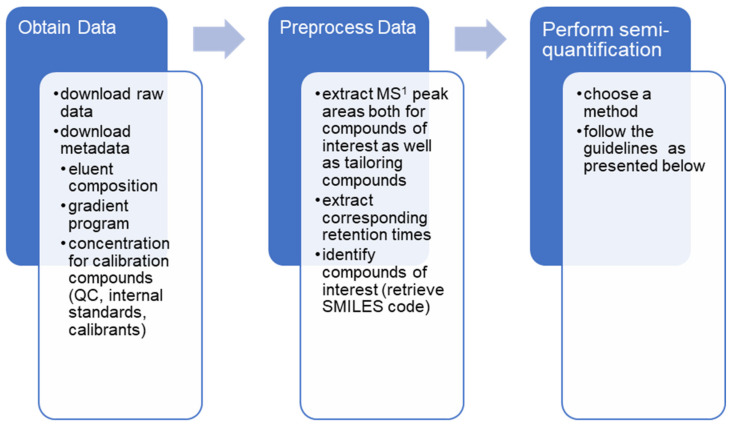
Performing retrospective analysis generally requires three main steps: acquire the data, data pre-processing and applying semi-quantification.

As mass spectrometric signal varies somewhat between days, and a lot between laboratories, semi-quantification open ways to better evaluate time series behavior in samples, or directly compare results of large cohorts measured by multiple labs. Furthermore, for the wider community, it is not only important to know what is in the sample, but also how much of it there is. Thus, semi-quantification combined with already collected data from repositories can reveal a lot of new, useful information. A general scheme of how to perform retrospective semi-quantification is presented in Figure 5.

Our experiences show that retrospective analysis is fully feasible if the necessary data are available. We have applied retrospective analysis on green tea samples originally measured at University of North Carolina, Greensborough, USA, in 2016 [71]. Semi-quantification of these samples was performed by us in the summer of 2018 at University of Tartu, Estonia. The retrospective semi-quantification became feasible because they measured reference standard, NIST green tea, together with the samples in the previous study. Reference standard with known concentrations is a great tool to use for calibrating the predictions to laboratory, matrix and method specific response factors. We were able to show that, using solely predicted ionization efficiencies for concentration estimation for polyphenols and similar natural products in green tea, the average error of predicted concentration was 1.9×. Moreover, in the former study, they detected and identified 5 additional compounds, for which they were lacking analytical standards. In our retrospective study, we could provide concentration estimates for those compounds as well.

## 5. Carrying Out Semi-Quantitative NTS in Practice

Generally, steps taken during the non-targeted analysis directly influence the accuracy of the semi-quantification. We suggest generic chromatographic method alongside a minimal sample preparation method to not lose compounds. However, doing analysis in both positive and negative mode as well as analyzing dilutions together with replica can significantly improve the accuracy. A step-by-step summary with notes is brought in Table 3. 

## 6. Conclusions

To aid in decision making, non-targeted LC/ESI/HRMS analysis together with methods for semi-quantifications are increasingly used. In this tutorial, we have presented appropriate NTS workflows and ready-to-use semi-quantification approaches. To avoid high errors related to the estimated concentrations, our overall recommendation is to use as general methods as possible throughout the entire analysis. 

We suggest doing as little sample preparation as possible, both to prevent losing compounds in the process but also to avoid poor recoveries of compounds. If possible, the best sample preparation is simply no sample preparation, however, if it is needed it is best to use generic methods. During analysis, it is recommended to run the samples in both positive and negative ESI mode to increase the number of detected compounds. If it is feasible, best results are achieved when using different mobile phases for ESI+ and ESI-: suggested mobile phases are presented in Section 2.2 and in Table 3, together with a generic gradient. We also propose to run the same sample at different dilutions, at least two but preferably more, to determine that the analysis is in the linear range by comparing the estimated concentrations with the dilutions. Before applying semi-quantification, it is important to account for fragmentation occurring in the ion source, especially if the sample might contain compounds that easily fragment, e.g., phthalates, carboxylic acids. In our experiences, we have seen that semi-quantification strategies based on ionization efficiency predictions give more accurate results. Therefore, we encourage the use and further development of such approaches. However, in the case of no tentative structures of the unknown compounds, only one semi-quantification strategy presented here is applicable, namely the closest eluting compound approach. 

In conclusion, we cannot stress enough the need of using generic methods and parameters, as suggested in Table 3, as well as the benefits of unifying the methods across communities, when doing NTS.

## Figures and Tables

**Table 1:** Structurally similar compounds do not necessarily mean that they have similar chromatographic properties. Here we see atrazine and some known TPs with corresponding score on the similarity to atrazine (parent) obtained from the online tool from University of Athens [19] and the RF measured in our laboratory. Chemicalize was used for prediction of p*K*_a_ and log*P*, July 2020, https://chemicalize.com/ (accessed on 9 June 2021) developed by ChemAxon (http://www.chemaxon.com) (accessed on 9 June 2021) [26].

Compound	Similarity Score	p*K*_a_	log*P*	RF
Atrazine 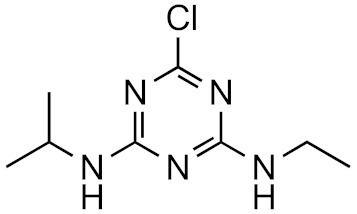	100%	4.2	2.2	7.3 × 10^17^
Atrazine-2-hydroxy 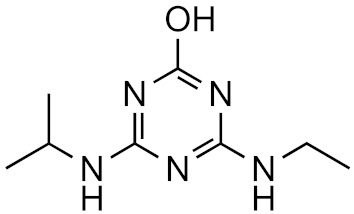	58.3%	3.6	−3.1	1.0 × 10^17^
Atrazine-desethyl 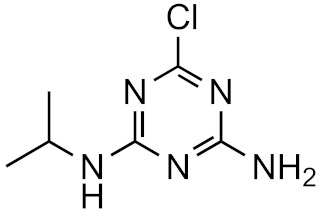	53.9%	4.4	0.8	4.1 × 10^16^
Atrazine-desethyl-2-hydroxy 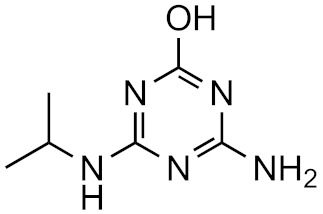	38.9%	3.0	−3.5	3.0 × 10^16^
Atrazine-desisopropyl 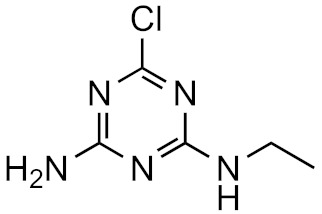	44.6%	4.4	0.4	2.7 × 10^16^
Atrazine-desisopropyl-2-hydroxy 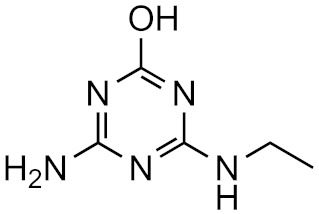	31.5%	3.1	−3.9	4.6 × 10^16^
Atrazine-desethyl-desisopropyl 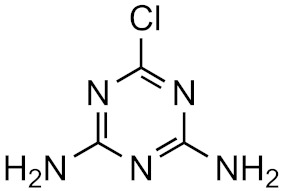	19.8%	4.6	−0.2	4.9 × 10^15^
Atrazine-desethyl-desisopropyl-2-hydroxy 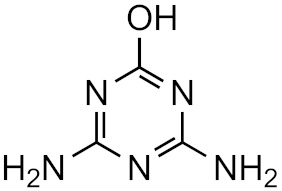	13.4%	3.1	−4.5	4.0 × 10^14^

**Table 2:** The closest eluting compound is in some cases not the same in different mobile phases, as is the case for valsartan and metsulfuron-methyl. Here we see how the retention time change causes the change of closest eluting compound and how this can affect the accuracy of the response factor prediction in ESI+.

Compound	pH	Neighbour	RT_compound_ (min)	RT_neighbour_ (min)	RF_compound_	RF_neighbour_	$\frac{RF_{compound}}{RF_{neighbour}}$
Valsartan 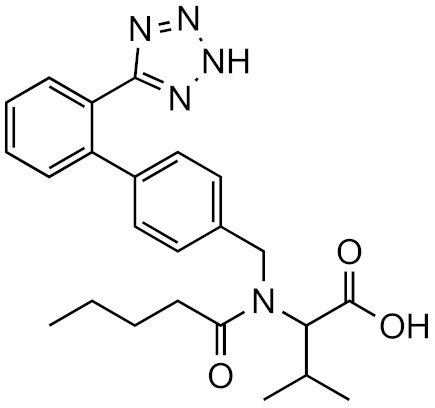	2.7	Estrone	12.58	12.58	5.99 × 10^11^	1.11 × 10^12^	0.54
	5.0	Chlorthalidone	7.30	7.41	1.52 × 10^11^	9.42 × 10^10^	1.61
	8.0	Sulfamethazine	5.23	5.27	7.93 × 10^11^	4.77 × 10^12^	0.166
Metsulfuron-methyl 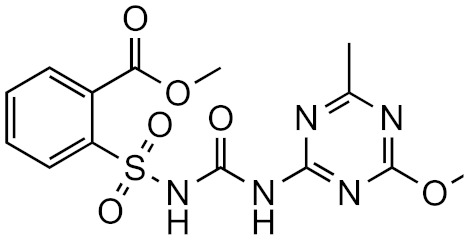	2.7	Prometryn	10.32	10.26	4.31 × 10^12^	6.23 × 10^12^	0.692
	5.0	2-napthoic acid	9.62	9.62	2.17 × 10^12^	2.53 × 10^9^	857
	8.0	Gabapentin	3.78	3.75	5.80 × 10^12^	2.52 × 10^12^	2.30

**Table 3:** An overview of the steps in NTS together with the possible impact on semi-quantification accuracy and measures to improve this accuracy.

NTS Step	Suggested Procedure	Reasoning
**Sample Preparation**	Avoid extensive sample preparation where possible.	If you analyze fairly simple liquids like water samples, urine, beverages, etc., direct injection of the sample is suggested over sample clean-up to avoid losses of analyte. Matrix effect can be evaluated by comparing the results from different dilutions, see below.
**Standards**	Prepare a solution with a set of compounds with known concentrations and analyze this set together with your samples. A suggested set of compounds could be tetrahexylammonium salt, haloperidol, diphenyl phthalate, tetraethylammonium salt, phenylalanine, dimethyl phthalate, progesterone, alanine, uracil, and saccharin for positive ESI mode. In negative mode we suggest 4-aminobutyric acid, sorbic acid, vanillin, benzoic acid, salicylic acid, p-nitrophenol, 3-nitrobenzenesulphonamide, perfluorobutyric acid, tetradecanoic acid, 3,5-diiodosalicylic acid, perfluorooctanesulfonic acid [32].	Make sure that these compounds cover a wide ionization efficiencies range and elute over the full chromatographic run. These compounds will be used to transfer the ionization efficiency predictions to your instrument scale.
**Sample Analysis**	Analyze samples on at least two dilutions.	Additional to running duplicates or triplicates, you can also analyze your samples at different dilutions or with different injection volumes. This will allow you to assure that all measurements are in the linear range as well as account for possible matrix effect. Matrix effect [72] is known to be less severe for more diluted samples.
**Chromatography**	Use generic chromatographic parameters. Avoid exotic additives and organic solvents. To generalize these methods, we recommend using 0.1% formic acid in ESI positive mode and ammonium hydroxide or ammonium formiate pH = 8.0 in ESI negative mode. In both cases a linear gradient from 5 to 100% of acetonitrile over 15 min for a 10 cm C18 column with 3 µm particle size has proven generic.	The predictions of any model are applicable only to the conditions used in the training/validation of the model. Therefore, rare LC conditions are likely not to be covered by the quantification model used.
**Ionization Conditions**	Choose soft ionization conditions, use the default parameters of the vendor as guide. If possible, do not alter these parameters too much. Run in both positive and negative ESI mode.	Soft conditions are likely to cause less fragmentation. The units and range of values of source parameters depend on the vendor, so exact parameters cannot be transferred between instruments. However, using vendor recommendations across instruments yields similar relative ionization efficiency values, and therefore, semi-quantification results [73]. Running analysis in both positive and negative mode enables combining and comparing results for polyfunctional compounds ionizing in both modes.
**MS Parameters**	Use a wide scan range, e.g., from 100 to 1000 Da.	Wide scan range will enable pinpointing fragments formed in the ionization source.
**Data Processing**	Combine the signal of the precursor ion and fragments together. Check that the integrations is acceptable.	Fragmentation occurs separately from ionization and is not accounted in the prediction algorithms. Poor integration of tailing or split peaks may significantly decrease semi-quantification accuracy.

## Data Availability

The data were available from the authors. The code is available from: https://github.com/kruvelab/NTS-semi-quant_review/ (accessed on 9 June 2021).
